# Health, Work, and Family Strain – Psychosocial Experiences at the Early Stages of Long-Term Sickness Absence

**DOI:** 10.3389/fpsyg.2021.596073

**Published:** 2021-03-30

**Authors:** Martin I. Standal, Vegard S. Foldal, Roger Hagen, Lene Aasdahl, Roar Johnsen, Egil A. Fors, Marit Solbjør

**Affiliations:** ^1^Department of Psychology, Faculty of Social and Educational Sciences, Norwegian University of Science and Technology, Trondheim, Norway; ^2^Department of Public Health and Nursing, Faculty of Medicine and Health Sciences, Norwegian University of Science and Technology, Trondheim, Norway; ^3^Department of Psychology, Faculty of Social Sciences, University of Oslo, Oslo, Norway; ^4^Research Institute, Modum Bad, Vikersund, Norway; ^5^Unicare Helsefort Rehabilitation Centre, Rissa, Norway; ^6^General Practice Research Unit, Department of Public Health and Nursing, Faculty of Medicine and Health Sciences, Norwegian University of Science and Technology, Trondheim, Norway

**Keywords:** mental disorders, musculoskeletal disorders, burnout, return to work, work-family conflict, work-life balance, social identity, long-term sick leave

## Abstract

**Background:**

Knowledge about the psychosocial experiences of sick-listed workers in the first months of sick leave is sparse even though early interventions are recommended. The aim of this study was to explore psychosocial experiences of being on sick leave and thoughts about returning to work after 8–12 weeks of sickness absence.

**Methods:**

Sixteen individuals at 9–13 weeks of sick leave participated in semi-structured individual interviews. Data was analyzed through Giorgi’s descriptive phenomenological method.

**Results:**

Three themes emerged: (1) energy depleted, (2) losing normal life, (3) searching for a solution. A combination of health, work, and family challenges contributed to being drained of energy, which affected both work- and non-work roles. Being on sick leave led to a loss of social arenas and their identity as a contributing member of society. Participants required assistance to find solutions toward returning to work.

**Conclusion:**

Even in this early stage of long-term sick leave, sick listed workers faced complex challenges in multiple domains. Continuing sick leave was experienced as necessary but may challenge personal identity and social life. Those not finding solutions may benefit from additional early follow-up that examine work-related, social and personal factors that influence return to work.

## Introduction

Long-term sickness absence is a significant challenge in industrialized countries (Gabbay et al., 2011). It is costly for society, and longer sickness absence is associated with adverse health outcomes, multimorbidity and an increased risk of permanent disability for individuals (Gjesdal et al., 2004; Waddell and Burton, 2006). Varied definitions of long-term sick leave exist, with a threshold usually between 4 and 8 weeks. For instance, the National Institute for Clinical Excellence in the United Kingdom define long-term sick leave as sick leave spells lasting more than 4 weeks (Gabbay et al., 2011). In Norway, 8 weeks is often used as work-related activity is demanded by legislation at this point (National Insurance Act, 1997).

Due to the negative impacts of long-lasting sick leave, early RTW interventions are recommended (Waddell and Burton, 2006; Black, 2008; Norwegian Ministry of Labour and Social Affairs, 2016). After the first few months individuals have a reduced relative likelihood of returning to work (Black, 2008; Norwegian Labour and Welfare Administration, 2019) and interventions between 8–12 weeks of sick leave are proposed to be a sensible approach order to help sick-listed individuals RTW (van Duijn et al., 2010; Hoefsmit et al., 2012; Aasdahl and Fimland, 2020). Furthermore, as many prognostic factors for RTW are shared across diagnoses, the process of returning to work has been argued to be a general process that contain elements that are similar across different health issues (Gragnano et al., 2018). However, attempts at finding effective cross-diagnostic interventions have resulted in inconsistent conclusions (van Vilsteren et al., 2015; Vogel et al., 2017; Aasdahl et al., 2018b; Cullen et al., 2018). Such difficulties in finding effects could be due to the multifaceted problems that sick-listed individuals face, which include interactions between the individual and other stakeholders such as the workplace, health services and social surroundings (Loisel et al., 2005). This means that length of sick leave is not only influenced by health status, but also psychological and social dimensions such as individual expectations, perceptions, as well as family life, work environment and workplace support (Landstad et al., 2009; van Vilsteren et al., 2015; Aronsson et al., 2017; Nigatu et al., 2017; Nilsen et al., 2017).

The importance of psychosocial factors in the RTW process are also demonstrated in findings that stressful family situations and a demanding work life are common experiences among long-term sick-listed individuals (Sandmark and Renstig, 2010; Frederiksen et al., 2015; Brataas and Evensen, 2016; Corbière et al., 2016). Meta-syntheses of qualitative RTW research also point to the disruption of social life during sick leave, stressful work situations, and the importance of supportive workplace for RTW, as common experiences (Froud et al., 2014; Thisted et al., 2018; Grant et al., 2019). The theory of role strain states that different obligations for the various social roles (e.g., at work, family, leisure) may not be compatible (Goode, 1960). In modern society these work and non-work roles converge and impact each other (Ford et al., 2007) and lack of support in these arenas can lead to conflict between these roles (French et al., 2018).

Furthermore, being on sick leave influence an individual’s identity and social roles (Gahnström-Strandqvist et al., 2003; Lännerström et al., 2013; Froud et al., 2014; Blank et al., 2015). For example, societal and personal expectations of sick leave may also influence individual behavior when sick-listed. Such expectations were described by Parsons’ ‘sick role’ theory which illustrates how society has viewed illness behavior (Costa-Black et al., 2013). In this theory the sick individual is seen as having lost the capacity to do valued tasks, albeit not being responsible for falling ill (Parsons, 1991). This loss of capacity affects all of the individual’s roles at work and outside of work (Varul, 2010). Consequently, the person is exempt from their normal roles and obligations and enters a ‘sick role’ where their new obligation is to spend their time and effort to get well as quickly as possible (Parsons, 1991). Thus, withdrawing form society is the expected behavior to fulfill the sick role (Varul, 2010).

Professionals aiming to support RTW have expressed a need to understand sick-listed individuals’ broader life-worlds (Eftedal et al., 2017). Research on experiencing sick leave is extensive, but despite recommendations for early interventions qualitative research focusing on the early stages of long-term sick leave is scarce. Previous research has commonly been performed with undefined or varied sick leave length, or with participants sick listed for several months or years who might not recall their earliest months of sick leave (e.g., Lännerström et al., 2013). These experiences may not be well-suited to inform early RTW interventions. Research has also focused on specific genders, occupations or diagnoses (Ahlstrom et al., 2017; Nilsen et al., 2017). Individuals with different genders, diagnoses or occupations may have different experiences of sick leave (Elderkin-Thompson and Waitzkin, 1999; McInnis et al., 2015). However, as returning to work can be argued to be a general process across health issues it is also important to know which experiences might be shared, not least considering the large heterogeneity within and similarities between such groups (Hankivsky, 2012).

Cross-diagnostic RTW approaches have been described as promising and early interventions (e.g., after 8–12 weeks of sick leave) are increasingly recommended (van Duijn et al., 2010; Hoefsmit et al., 2012; Schultz et al., 2016; Aasdahl and Fimland, 2020). However, these fields are still in an early phase and there is a need to identify aspects of the RTW process that are common across disorders and contextualized by time (Schultz and Gatchel, 2016; Schultz et al., 2016). Thus, more information on common psychosocial experiences of being sick listed across diagnoses in the early stage of sick leave could inform early cross-diagnostic RTW interventions and follow-up.

The aim of this study was to explore psychosocial experiences of being on sick leave after 8–12 weeks of sickness absence in a heterogeneous sample. In particular, we examined:

Which experiences were shared among individuals at this stage of sick leave?
How did participants experience sick leave follow-up at this stage of sick leave?
What were participants’ thoughts and expectations about returning to work?

## Materials and Methods

This is a phenomenological interview study nested within a RCT evaluating the effect of motivational interviewing on sickness absence (Aasdahl et al., 2018a).

### Study Setting: Follow-Up of Sick-Listed Workers in Norway

In Norway, employees are entitled to 12 months of full wage benefits when on sick leave. The first 16 days of sick leave are paid by the employer and the rest is paid for by the National Insurance Scheme through the NAV (Norwegian Labour and Welfare Administration, 2020b). Sick leave is also encouraged to be graded, meaning that employees work a percentage corresponding to their current work ability.

The employer has the main responsibility for assisting the sick-listed worker back to work. Within the first 4 weeks of sick leave, the employer and sick-listed worker are obliged to create a plan detailing measures which can help the sick-listed RTW. Within 7 weeks, the employer is required to arrange a meeting with the sick-listed worker that may also include other relevant stakeholders. If work-related activities are not resumed within 8 weeks, an expanded medical certificate that documents medical problems preventing such activities is required. Before 6 months have passed, a mandatory dialogue-meeting must be arranged by NAV. This meeting includes the NAV caseworker, the employer, the sick-listed worker and, in some cases, the GP. The sick-listed worker can, at any time, request a meeting with a NAV caseworker or request a dialogue-meeting that also involves the employer (Norwegian Labour and Welfare Administration, 2020a).

### Recruitment and Participants

Eligible participants in the present study were workers aged 18–60 years living in Central Norway who were at 8 weeks of sick leave with a current sick leave status of 50–100% and any diagnosis. Eligible individuals were identified at 7 weeks of sick leave by NAV and invited to participate in the RCT through NAV’s electronic communication website. The list of participants who accepted were then forwarded to a researcher. The present interview study used a convenience sampling strategy where all those who had consented to participate in the RCT between November 2017 and February 2018 were eligible participants. In total, 73 individuals were contacted by one of the authors (MIS or VSF) via e-mail and invited to participate in an interview where they would talk about their experiences of sick leave. Thirteen women and three men with current sick leave length of 9–13 weeks and an age range of 32–59 participated in this study (see Table 1 for descriptive information). Fifty-seven individuals declined the invitation or did not respond to the e-mail. No researchers in the present study had any prior relationship with the participants.

**TABLE 1:** Participants descriptive information.

**Participant**	**Age**	**Gender**	**Self-reported illness**	**Education**	**Sector**
1	31–40	Female	CMD	Higher (>3 years)	Public
2	31–40	Male	CMD	Higher (3 years)	Private
3	51–60	Female	MSK	Trade school	Private
4	31–40	Female	CMD	Higher (3 years)	Public
5	31–40	Female	CMD	Higher (>3 years)	Public
6	41–50	Male	Other	Higher (>3 years)	Private
7	51–60	Female	MSK	Higher (3 years)	Public
8	51–60	Female	CMD	Higher (>3 years)	Public
9	41–50	Male	MSK	Higher (>3 years)	Private
10	51–60	Female	CMD	Trade school	Private
11	31–40	Female	CMD	Higher (>3 years)	Public
12	Missing	Female	CMD	Missing	Public
13	41–50	Female	Other	High school	Public
14	41–50	Female	CMD	Higher (>3 years)	Public
15	41–50	Female	CMD	Higher (>3 years)	Private
16	51–60	Female	Other	Higher (3 years)	Private

*Education is described as the participant’s highest completed education (higher education is at the university/college level). CMD, common mental disorder; MSK, musculoskeletal disorder.*

### Data Collection

Individual interviews were chosen, as they provided a safe space for rich, in-depth descriptions from each individual. Prior to inviting participants, we estimated the need for 10–15 interviews based on our research aim and the experience of the interviewers, guided by the concept information power (Malterud et al., 2016). There is little consensus regarding how to achieve saturation in phenomenology and the amount of information needed should be evaluated throughout the process (Malterud et al., 2016). Thus, the necessary number of interviews was evaluated consecutively from nine interviews based on dialogue quality and information obtained. We found that participants shared their stories willingly and that dialogue quality was acceptable to extract information that had relevance for the study aim, indicating adequate information power (Malterud et al., 2016). No thematically new information was obtained from the final three interviews, closing the data collection at sixteen interviews. The quality of information obtained from our informants was considered satisfactory for describing the experiences of the participants.

Interviews were performed at a university campus by one of the researchers (MIS or VSF). Written informed consent was obtained prior to the interviews. Each interview followed a semi-structured interview guide with five broad major questions (see Table 2) and allowed for follow-up questions when necessary. Interview questions were created in collaboration between all authors based a biopsychosocial understanding of sick leave. In biopsychosocial models sick leave length is not only influenced by health, but also psychosocial factors and involvement of the stakeholders in the RTW process (Loisel et al., 2005). Follow-up questions thus addressed personal attitudes and motivations and the context surrounding the sick listed worker such as experiences with regards to family, friends, work, co-workers, supervisors, the welfare system, their general practitioners and other healthcare services (see Supplementary Materials for the follow-up questions). All interviews were audio recorded and were transcribed verbatim.

**TABLE 2:** Interview guide.

Could you tell us about being on sick leave?
Could you tell us about the assistance that you have received during your sick leave?
What are your thoughts about returning to work?
What motivates you to return to work?
What is it like talking about being on sick leave?

### Analysis

Data was analyzed using Giorgi’s descriptive phenomenological method (Giorgi, 2009) which offers a method for gaining knowledge of a specific phenomenon, such as being on sick leave. Descriptive phenomenology attempts to understand how something is experienced from the perspective of the person undergoing the experience and is not interested in whether these experiences are true or false. The method thus allows for examination of the subjective experiences of individuals and how people create meaning in their situations (Giorgi, 2009). In order to describe the phenomenon as experienced by the participants, it is necessary for the researchers to reflect on and set aside preconceived assumptions, also known as bracketing (Giorgi, 2009). Preconceived assumptions were reflected upon individually and then discussed by all researchers in an early meeting to reduce their impact on data collection and analysis.

The analytic steps undertaken in this study were as outlined by Giorgi (2009):

1. Reading the complete interview transcript to get an overview of the individual’s situation.
2. Re-reading the transcript and breaking the data into parts by marking whenever there was a transition of meaning in the data, creating meaning units.
3. Transforming the data from meaning units into expressions that more generally described the issue in the meaning unit while still holding true to the specific situation.
4. Developing the theme(s) of the interview by organizing the expressions in the previous step. These themes are general expressions of the descriptions found in the interview.

These four steps were undertaken for all interviews by MIS and VSF (see Table 3 for an example) and themes were developed separately based on the meaning units. All other authors developed themes from two to four interviews each. Thus, all interviews were analyzed by at least three authors to reduce the impact of preconceptions from a single researcher. The themes from each interview were then discussed in group sessions with all authors. Sub-themes were developed from these discussions of data and further condensed into three major themes that described the situation for most participants in the study (see description of major and underlying sub-themes in the Supplementary Materials).

**TABLE 3:** Example of analysis process.

**Step 1 – An overview**
This individual is struggling with a demanding job, an illness that does not have a clear diagnosis and prognosis, and a lack of energy available for personal life.

**Step 2 – Creating meaning units**

*“Being able to take a step back and recover and not have to go ‘all-in.’ That I can relax at home and*… *gather energy until the kid comes home and I can spend the energy playing with him. Because just lying on the couch when he wants to play with you and you just doesn’t have the energy to do it. It’s really a stab in the heart.”*
///
*“And we didn’t really do anything either. I didn’t have the energy to get out of the house. So now there’s more I want to do, even though I can’t do everything.”*

**Step 3 – Transforming the meaning units to generalized expressions**

This individual feels (s)he has been able to recover energy that (s)he can spend with the child when on sick leave.

**Step 4 – Developing themes**

Energy depleted – work-family conflict

## Results

Three major themes emerged from the analysis: (1) energy depleted, (2) losing normal life, and (3) searching for a solution. The first two themes concerned participants’ experiences with being on sick leave, while the latter theme focused on their thoughts regarding returning to work. Each theme will be presented in detail below.

### Energy Depleted

Participants experienced a situation where a combination of health issues and work and family stressors were experienced as energy draining, and they contributed to an inability to function to the participants’ standards in either arena. Participants commonly struggled with symptoms such as pain, fatigue, dizziness, low blood pressure, memory or concentration problems:

*F 33: ‘So I’ve been dizzy since January, that’s my thing, but I didn’t get sick leave until May. [*…*] I used to come home from work exhausted [*…*], take care of the kid, and then just sleep until I got up for work the next day.’*

Being on sick leave was experienced as necessary to distribute their remaining time and energy to better manage their recovery and other simultaneous stressors. In their personal life, such stressors were often related to responsibilities toward their children. This could lead to challenges since the welfare system does not allow sick leave due to care responsibilities.

*F 39: ‘*… *this time I’m on leave to a larger degree because of my daughter, who is struggling with mental illness, [*…*] I’ve arranged with my GP that I can use a [burnout] diagnosis to be able to be home.’*

However, such stressful family situations on their own could also contribute to exhaustion and ill health. Experiences of overwhelming care responsibilities were not described by the male respondents, who rather described being able to better prioritize family life when sick listed.

Aspects of the participants’ work could also contribute to the perceived necessity of sick leave in their current situation. These aspects could include consistently having a high work load or adjusting to new energy-demanding work roles. Some individuals also disliked their job, which led to questions about whether to find a new job, but economic and social commitments made it hard to decide whether to stay or attempt to change jobs:

*M 38: ‘If you feel that there is something at the workplace that is difficult [*…*], the line of business is not right for you or the role you have is not right. [*…*]. This is not something that the GP or employer can fix, and when you have commitments with family and stuff, you can’t just leave to follow some kind of dream.’*

For those with less demanding family or work life, sick leave was nonetheless necessary in order to have energy left after treatment so they could function in their personal life while recovering. Without the capacity to do work, there was no use in them being at work:

*F 58: ‘the way I am now, I think it’s OK to be on sick leave because I’m ill and then it’s OK. [*…*] the disease makes me tired*… *a loss of concentration and capacity really. [*…*] so I need to rest and I can’t function at work so then it’s OK to be on sick leave.’*

Participants thus experienced sick leave as providing a necessary respite in an overwhelming situation. Sick leave provided the opportunity to prioritize their time and energy to better cope with their recovery and personal life.

### Losing Normal Life

Needing sick leave to resolve their problems was not without challenges. Working was important to the participants, as work was an arena where they felt appreciated and competent. Working made them feel that they contributed to their workplace, family and/or to society.

*F 59: ‘This is not a situation I like to be in. I enjoy being at work. I enjoy filling my days with something*… *so I know that I’m useful. So being on sick leave. but I have to*… *the way I am now, because I can’t go to work now. [The pain] will get worse if I’m at work.’*

Being sick listed thus challenged their identity as hard-working, contributing members of society, and they experienced an implicit expectation that they should go to great lengths to work even if they were sick. Sick leave was viewed as a last resort, and some now realized they should have started sick leave earlier:

*F 45: ‘I have a teenager that is acting out and it’s just a lot to handle right now [*…*] and if you’re up all night arguing with a teenager you can’t sleep at the same time. So then it’s completely unrealistic to go to work the next day. It’s not reasonable to go on like that and you just do a poor job. [*…*] so I probably worked a month longer than I should have. [*…*] the last month was horribly bad. I just wasn’t present at work.’*

Thoughts related to losing their identity as a contributing member of society also caused social challenges, as they wanted to appear normal despite feeling abnormal. Aspects such as uncertainty in diagnosis or prognosis could more readily be shared with their employers and colleagues, while other issues, such as unhappiness with their work or needing sick leave to care for a family member, were kept out of the conversations. Even when experiencing support, they were reluctant to talk too much about their situation, as it could be overwhelming for others and a negative focus. One had experienced such discomfort at a social occasion with colleagues:

*M 47: ‘I looked forward to the event [*…*] and there was a lot of nice conversation, but there was also a lot of talk about me and my neck, which is not what I wanted. [*…*] and people ask because they care [*…*] but it can be too much.’*

Participants also struggled with expressing why they sometimes felt fine, but at other times were exhausted:

*F 58: ‘I have friends that have asked how long I’m going to be on sick leave. And I tell them I don’t know but the doctors say it could be 2–3 months [*…*]. Then they get surprised because I look just fine and when I talk to them I sound fine. What they don’t see is when I’ve spent an hour or two with friends or some other small task, I’m stuck in my reclining chair for a couple of hours. They don’t see that.’*

Those who had few objective signs of illness or were having problems finding a clear diagnosis often felt a greater need to explain their situation. Some experienced that their explanations and reasons for sick leave were perceived as illegitimate. This led to avoiding activities, social events and neighbors in order to avoid questions about why they were out while being sick listed or why they were at home and not at work during the daytime. Some self-imposed such ideas:

*F 33: ‘No one is preventing me*… *I mean, my illness may prevent me, and I need to take that into consideration, but neither my employer or NAV or anyone is limiting me [*…*]. That’s not the problem when I go to [a concert]. It is allowed, actually, but no*… *I just feel uncomfortable, socially speaking.’*

For those who enjoyed their work environment, sick leave caused them to lose work as an arena for social interaction. This was added to the social avoidance behavior outside of work and contributed to an experience of being excluded:

*F 33: ‘When I’m on sick leave, I don’t have the energy to meet people because I kind of have to focus on getting better. [*…*] So I’ve gone from being very involved at work to not knowing anything about what’s happening, and I feel like that’s an extra burden as well.’*

Thus, being on sick leave challenged the participants’ identity and impeded their social life, which contributed to the feeling of being abnormal.

### Searching for a Solution

Participants needed assistance in order to find solutions to resolve their challenges and progress toward returning fully to work. All participants described much effort through examinations and treatment from their GP and healthcare services. However, some expected a faster recovery than what was reality.

*M 47: ‘I didn’t realize that the pain that day would side-line me for so long. I thought I might get an hour or two with the chiropractor and then I’m done. Do some stretches*… *but it didn’t work that way.’*

Finding a diagnosis that explained their symptoms could also be challenging. This caused uncertainty as to how they were going to make progress.

M 38: ‘I’m at the hospital and we do x-rays, we do ultrasound, we do all the tests and everything is OK. The heart is fine, there is no hormonal imbalance, no Lyme disease, so I’m left here after months of examinations with nothing. And I ask my GP: What do I do? I want to be well, I want to function, so what’s the deal?’

Lacking a solution also contributed to a feeling that returning to work was dependent on factors outside of their control. Such factors included improvement related to their child’s situation, figuring out what was causing their own symptoms or a sudden improvement in their health:

*F 39: ‘It’s difficult to say when*… *it depends on [what happens to my daughter]. It’s not about how I feel, because I feel pretty OK now you know.’*

*F 35 ‘*… *my diagnosis is one that may pass just as fast as it came. Just by doing exercises and relaxing, and then you can wake up the next morning and be well. It has not happened yet, but we are hoping.’*

In addition to health services, assistance from their employers was also important. The employer could assist through emotional support and by accommodations at work, such as changed responsibilities or flexible work hours. This helped to reduce the uncertainty of whether they could cope with the expected work demands and reduced the threshold of whether they would attempt a RTW.

Nearly all participants were aware of the negative aspects of social isolation and inactivity in sickness absence. In cooperation with their GP, they tried to remain active and not lose contact with their workplace. Graded leave was common, and using graded sick leave allowed them to keep in contact with their workplace, test their work capacity and to have enough energy left to function outside of work. Graded sick leave was also used to normalize their situation and alleviate some of the social stigma:

*M 38: ‘I’m thinking, I have to be home. I’m sick. I can’t go out and have a burger and a beer with a buddy because that might look bad. Those from work may not understand that I’m generally not well, but I might have a good day. [*…*] “if you’re well enough to go out, you’re well enough to be at work.” [*…*] this is one of the reasons why I want to be on graded leave, because then I can live more normally.’*

Employers were mostly experienced as being supportive, as they largely let the sick-listed individuals determine the RTW pace and attempted to adjust their work tasks in order to fit graded sick leave. When determining the RTW pace, the sick-listed individual’s focus was finding a pace that balanced work, health and personal life.

*F 50: ‘When I’m going to start to work 100% again, I think I need a deal with my employer and my GP that if I’m coming home from work exhausted and can’t do anything*… *then I think it’s too early to start 100%. Because I’m going to have a life outside of work, too*… *and we need to see how we can work that out.’*

Fear of a more difficult situation due to returning to work too quickly was a major concern. Uncertainty in their work ability led to worry that if they returned to work faster than they could manage, it could result in a worsening of their health or their personal situation:

*F 35: ‘*…*knowing that I’m going to be [at this job] for many years contributes to being able to take this time off and make my head work again. [*…*] and really get well, not just going to work [sooner] and becoming worse. And then it will take even longer.’*

If the job inherently contained undesirable tasks, or if the sick-listed individual was unhappy at work, they realized that there was little the employer could do.

*M 38: ‘I’ve thought about this [*…*] maybe I should do something that is more meaningful for me. [*…*] Instead of trying to sell as much stuff as possible, maybe do something that can help people. [*…*] I don’t know how the employer can make adjustments for my situation, because here we are talking about the line of work you are in.’*

The assistance the sick-listed workers received from NAV consisted of a standardized letter informing them of their duties and rights during sick leave. Some were surprised by this and stated that they expected more contact. Most wondered how NAV could know if they were progressing toward RTW. They did, however, differ in their perceived need for help from NAV. Finding a solution in order to make progress toward returning to work was not always easy. Those who did not find solutions from other services and were uncertain about how to make progress generally expressed more need for NAV involvement, but only one individual initiated contact on her own. Others viewed early sick leave as a situation where the sick-listed, their GP and employer are in control and suggested that NAV should not interfere.

## Discussion

This study explored psychosocial experiences with sick leave and thoughts about returning to work among individuals with 9–13 weeks of sick leave. The results demonstrate the multidimensional and inter-connected nature of sick leave experiences, also at an early stage of long-term sick leave. In addition to health issues, challenges related to work and family life also contributed to the need for sick leave. Sick leave had a negative impact on participants’ identity and social life, but was viewed as necessary in order to distribute their energy to resolve their challenges. RTW was desirable, but depended on their health and overall situation, and participants needed assistance in order to find solutions that would help them progress toward returning fully to work.

For participants in the current study, a combination of health issues and family or work stress contributed to a situation where they felt drained of energy, and this made functioning outside of work incompatible with recovery and working. The theory of role strain describes the difficulty of fulfilling role obligations due to excessive strain (Goode, 1960). Experiencing pressure to devote time and attention beyond one’s capabilities to a single role obligation will increase strain for the individual (Goode, 1960). According to the negative emotional spill-over effect, stress at work can lead to negative feelings, such as worry, doubt, disappointment and frustration, that spill over into private life and make it difficult to pursue a satisfying non-work life (Evans and Bartolomé, 1984). On the other hand, demands in private life also influence work and health, but have received less attention. For example, individuals might experience increased strain due to illness or responsibilities in their family that affect time and energy available for the other arenas in their life (Kosny et al., 2018).

Hamnes et al. (2017) interviewed sick-listed individuals who experienced a gradual opting out of other arenas to the point where life only revolved around working, fatigue and resting, finally resulting in sick leave. These findings are aligned with the results in the present study which indicated sick leave was needed to adjust the balance between self-care, family and recovery. These aspects were prioritized over work because sick leave is only possible in the work arena. Reducing a rewarding work role to compensate for demanding personal lives might not be an optimal solution. However, withdrawal from the demands of the family role is difficult and may cause feelings of guilt, as well as pressure from others (Goode, 1960). Hamnes et al. (2017) also found that individuals chose to work fewer hours, worked part-time and attempted to reduce out of work stressors in order to achieve a better balance between work, family, social life and physical activity. This could be a similar, albeit more long-term, strategy as compared to the need for sick leave in the present study.

Participants had mixed feelings toward being on sick leave. As described above, sick leave was experienced as necessary to redistribute remaining energy to improve their situation, but sick leave also led to social avoidance behavior and a feeling of being ‘abnormal.’ Working can be viewed as a signal to others that one is normal and beneficial to society (Hamnes et al., 2017) and sustainable work participation has also been closely linked to experiencing a meaningful life (Klevanger et al., 2018). Disruption of roles that are important to one’s self-image may cause individuals to feel ‘lost’ (Goode, 1960), and illness may lead to loss of self-image and social isolation (Charmaz, 1983). This highlights the identity-bearing aspects of work as well as work as a social arena.

Prolonged sickness absence has also been found to change the sick leave experience from a necessary opportunity to rest toward a negative circle of pain, inactivity and isolation (Ockander and Timpka, 2001). The sick role theory assumes that the withdrawal behavior that is expected when on sick leave has an impact on all of the person’s role performances and prevents the possibility to receive appreciation from other arenas (Varul, 2010). Withdrawal behavior may also lead to social isolation and restrict activities that could promote recovery (Charmaz, 1983). For several in the present study, it was easier to avoid social situations which they felt required explanations for their participation. Behaviors that did not appear to promote their own well-being (e.g., socializing while being sick listed) may cause conflict to the expected behavior of the sick role. This could be reinforced by having an illness invisible to their surroundings, making the decision to disclose their illness difficult and stressful, with the potential consequences of being rejected and stigmatized (Joachim and Acorn, 2000). However, most individuals in the present study realized that lack of activity, social isolation and distancing themselves from their workplaces might obstruct their recovery and RTW. Withdrawing from work obligations may be experienced as necessary, but Parsons’ theory of the sick role suggests that legitimate absence from work includes retreat from other roles (Varul, 2010). Such total withdrawal is problematic as social isolation may be a predictor of prolonged absence (Steenstra et al., 2005). Recent research has also indicated that freedom from the ‘sick role’ can be an important part of recovery for patients with chronic illness (Cheshire et al., 2021). RTW professionals may thus have an important role in reinforcing health promoting behaviors when such behaviors appear to contradict what is expected in the sick role.

Moreover, the social insurance system limits how long sick listed workers can withdraw from their work obligations. In Norway, sick listed workers are required to take part in work activities after 8 weeks of absence or obtain an expanded medical certificate (Norwegian Labour and Welfare Administration, 2020c). Parsons describe a moral obligation to overcome the sick role as soon as possible (Varul, 2010). By demanding work activities, the system turns this moral obligation into a structural obligation, essentially deterring continuing withdrawal. For many sick listed in the present study, this was problematic as they experienced returning to work outside of their control. Thus, in order to find solutions and progress toward returning to work, participants needed assistance, for instance from the employer or their GP. In the present study, employers were largely willing to adjust work and facilitate graded leave, which can promote RTW (van Vilsteren et al., 2015). However, not all reasons for absence were conveyed to the employer. For instance, proper adjustments may not be possible when absence is partly due to dissatisfaction with the job or stress due to childcare. Increased co-worker load due to sick listing or a reason for sick leave that lacked apparent legitimacy can create tension with colleagues (Tjulin et al., 2011). On the other hand, communicating limitations reduces co-worker resentment (Skivington et al., 2016). Thus, there is a difficult trade-off in the balance of transparency and confidentiality when deciding how much to share. When experiencing a conflict between work and personal life, there is an imbalance in whether this conflict is deemed acceptable. Kelloway et al. (1999) argued that keeping personal life out of the workplace is an established norm, thus spending time and effort on private roles while at work is rarely acceptable. On the other hand, thinking about work in private life or disrupting family plans, such as having to work late, is more acceptable (Kelloway et al., 1999).

Participants were apprehensive about prioritizing their RTW, but rather viewed it as a consequence of better health or the resolution of their other challenges. Using graded leave enabled a balance between the benefits of returning to work and the fear of a worse situation due to returning to work. Re-entering work through graded leave helps the individual to comply with the expectations of the sick role while restoring some normality of everyday life. Returning to the workplace while still undergoing treatment, and before full recovery, has been described as important in returning to work (MacEachen et al., 2006). Graded leave could also have a positive effect on RTW and sustainable work participation (Markussen et al., 2012). However, this may partly be due to health selection effects, as less healthy individuals might be unable to work at all (Nossen and Brage, 2013). Apart from assistance from healthcare services and *ad hoc* employer assistance, participants had not experienced any structured RTW follow-up during the first few months of sick leave. This could be problematic for expedient RTW since other stakeholders see the GPs as a large contributor toward the patient’s RTW, while the GPs view themselves primarily as advocates for their patients, their well-being and health (Mazza et al., 2015). Thus, for some there may not be a stakeholder present focusing on RTW at this stage. The importance of early workplace involvement is emphasized in RTW best practice suggestions (Kristman et al., 2020) and those not experiencing sufficient assistance from an employer may have an increased need for co-operation with social insurance caseworkers (Eftedal et al., 2017). As many of the participants in this study expected some form of contact with social insurance services, caseworker assistance might be useful in order to facilitate and coordinate the RTW process among the sick-listed individual, their GP and their employer.

### Strengths and Limitations

A particular strength of this study is its descriptions of early sick leave using open-ended questions. Moreover, the data was analyzed by researchers from varied backgrounds (psychology, sociology, medicine), contributing to diversity in analytical discussions. Researcher triangulation is important to promote rigor in qualitative research by providing checks and verification of the research process (Elliott et al., 1999; Pitney, 2004). In this study, researcher triangulation was used at all stages of design, data-collection, analysis and writing of the manuscript.

A limitation of this study is its low recruitment rate. In this qualitative study, we invited 73 of the participants in the RCT and 16 agreed to participate in interviews. This could have led to a selection bias where other kinds of experiences with sick leave are missing from our data. For instance, women and individuals with higher education may be overrepresented in the current sample. Using a purposive sampling strategy to recruit a more homogeneous sample or a different gender balance could have resulted in other descriptions based on different experiences. Also, both interviewers were male with a psychology background, which could have affected interview responses. More variability in interviewers (gender, age, background) might have allowed other responses. These limitations can hinder the transferability of the results which refer to the ability to transfer experiences and results to situation with similar characteristics (Pitney, 2004). However, we have given rich descriptions of the data and reported the context of the study which allow the reader to determine whether the results speak to their situation and can thus heighten transferability (Elliott et al., 1999; Pitney, 2004). Nonetheless, to increase confidence in the results this study needs conceptual replication in other samples, such as those including more lower educated workers and more men, in order to examine whether similar or different cross-diagnostic experiences can be identified.

### Practical Implications

The results in this study show that experiences previously identified among long-term sick-listed such as demanding work and personal life (Thisted et al., 2018), social disruption (Froud et al., 2014), and uncertainty in balancing illness and RTW considerations (Andersen et al., 2012; Froud et al., 2014; Grant et al., 2019), were also relevant across health concerns at an early stage of long-term sick leave. These experiences demonstrate the complex and multidimensional aspects of sick leave, which could mean that multiple stakeholders with different perspectives may be needed in early interventions to assist sick-listed individuals back to work. Expectations of caseworker involvement at this stage could indicate that individuals who struggle to find solutions will welcome an outside perspective to their situation, beside what their GP or employer provides. Caseworker involvement at an early stage could facilitate early identification of psychosocial barriers to RTW, such as uncertainty in RTW and disruption to social life. For instance, in a recent study we found that early caseworker involvement contributed to building confidence in the sick-listed’s RTW plan and normalized the sickness absence, which reduced the experience of guilt and stigma (Foldal et al., 2020). Further research should thus investigate the potential for social insurance caseworkers to identify and provide additional assistance to those with psychosocial barriers to RTW that are not easily captured by healthcare services or employer at this early stage of long-term sick leave.

## Conclusion

Multiple simultaneous challenges regarding health issues, work- and personal stressors are experienced by sick listed workers already at an early stage of long-term sick leave. When continuing sick leave is necessary to resolve these concerns, individuals on sick leave experience expectations that one should withdraw from society to focus on recovery. Such withdrawal is problematic for the identity and social life of sick listed individuals, and inactivity is often counter-productive for fast RTW. Sick listed workers thus face a difficult dilemma between returning to work to restore normal life and the perceived necessity of continuing sick leave. At this stage of sick leave healthcare services and employers are the main stakeholders involved in the RTW process. However, solutions to personal challenges may be outside the reach of these stakeholders. Thus, those individuals who are struggling to find solutions to their challenges could benefit from individually tailored additional early follow-up that proactively examine work-related, social and personal factors that influence RTW.

## Data Availability Statement

The datasets presented in this article are not readily available because “The datasets generated and analyzed during the current study are not publicly available due to protecting the anonymity of participants.” Requests to access the datasets should be directed to MS, martin.standal@ntnu.no.

## Ethics Statement

The studies involving human participants were reviewed and approved by Regional Committees for Medical and Health Research Ethics in South East Norway (No: 2016/2300). The patients/participants provided their written informed consent to participate in this study.

## Author Contributions

RH, LA, RJ, and EF designed the overall study. MSt, VF, and MSo contributed to the design of the interview study. MSt was in charge of writing the article. All authors contributed to development of the interview guide. MSt and VF did all interviews. MSo supervised the data collection. MSt and VF read and coded all interviews. RH, LA, RJ, EF, and MSo read and coded two to four interviews each. All authors contributed in sessions on analysis and developing the results, contributed to writing the article, and approved the final version.

## Conflict of Interest

The authors declare that the research was conducted in the absence of any commercial or financial relationships that could be construed as a potential conflict of interest.

## Abbreviations

GP: general practitioner
NAV: Norwegian Labour and Welfare Administration
RCT: randomized controlled trial
RTW: return to work.
