# Supplementary material for: Health, Work, and Family Strain – Psychosocial Experiences at the Early Stages of Long-Term Sickness Absence
Source: Front Psychol. 2021 Mar 30;12:596073. doi: 10.3389/fpsyg.2021.596073 (PMC8043853; doi:10.3389/fpsyg.2021.596073)
Supplement: Supplementary file 1 [file Table_1.DOCX]

Supplementary table – Interview guide with follow-up questions

**Could you tell us about being on sick leave?**Ask about sharing any experiences.
Ask about contact with others (family, friends, neighbors, colleagues or others in your circle).
Ask about positive or negative aspects of being sick listed.

**Could you tell us about the assistance that you have received during your sick leave?**Ask about experiences and expectations from GP, healthcare services, employer, NAV/social insurance services.
Ask about anyone else that have been helpful while sick listed.
Ask about experiences of cooperation between these stakeholders.
Ask about potential improvements for these stakeholders to help individuals return to work.

**What are your thoughts about returning to work?**
Ask about expectations (from oneself, employer, family, others).
Ask about facilitators and barriers.
Ask about what is demanded of you (from oneself, employer, family, others).
Ask about graded leave.

**What motivates you to return to work?**Ask about motivational factors (colleagues, social life, money, health, pressure, demands).

**What is it like talking about being on sick leave?**Ask about talking about sick leave in everyday life.
Ask about how the situation/sick leave is presented to others (family, friends, healthcare, NAV, employer, colleagues).
Ask about to whom being on sick leave is (not) talked about.
