# Supplementary figures and images for: Health, Work, and Family Strain – Psychosocial Experiences at the Early Stages of Long-Term Sickness Absence
Source: Front Psychol. 2021 Mar 30;12:596073. doi: 10.3389/fpsyg.2021.596073 (PMC8043853; doi:10.3389/fpsyg.2021.596073)

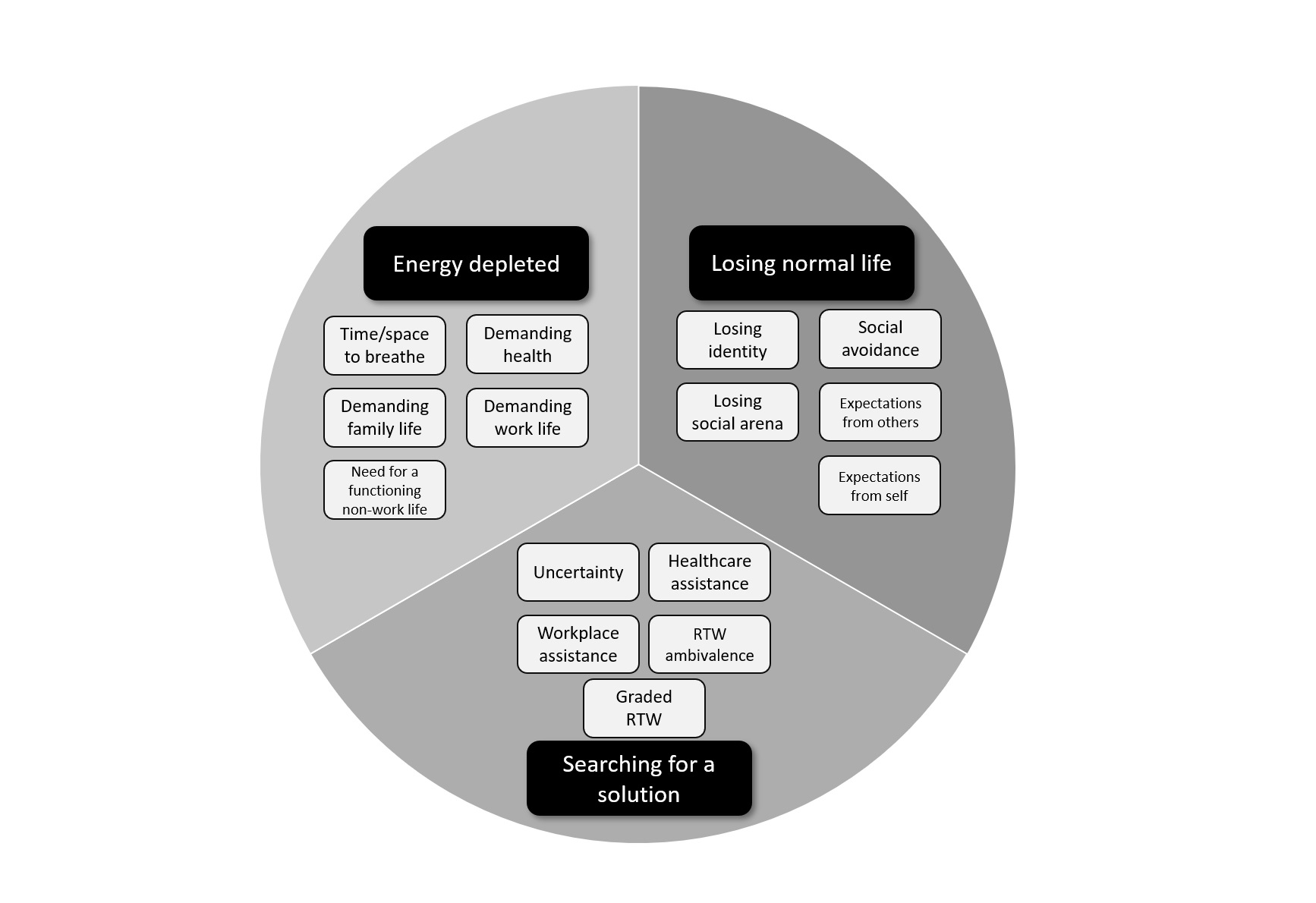

Supplement: Supplementary file 2 [file Image_1.JPEG]
